# The emotional equation: how psychosocial support boosts safety practices in the context of construction 5.0

**DOI:** 10.3389/fpsyg.2025.1632848

**Published:** 2025-08-18

**Authors:** Lei Zhang, Jingfeng Yuan, Xianfei Yin, Tiantian Gu, Yinghao Lu, Ping Liu, Mirosław Skibniewski

**Affiliations:** ^1^Department of Intelligent Construction and Management, Nanjing Tech University, Nanjing, China; ^2^Department of Construction and Real Estate, Southeast University, Nanjing, China; ^3^Department of Architecture and Civil Engineering, City University of Hong Kong, Hong Kong, China; ^4^China University of Mining and Technology, Xuzhou, China; ^5^Department of Data Science and Big Data Technology, China Agricultural University, Beijing, China; ^6^School of Civil Engineering, Lanzhou University of Technology, Lanzhou, China; ^7^Department of Civil and Environmental Engineering, University of Maryland, College Park, MD, United States; ^8^Institute for Theoretical and Applied Informatics, Polish Academy of Sciences, Gliwice, Poland; ^9^Chaoyang University of Technology, Taichung, Taiwan

**Keywords:** construction 5.0, safety practice, psychosocial support, safety participation, leadership safety behavior

## Abstract

**Background:**

Construction 5.0, which emphasizes human-centric technologies and improved collaboration between humans and machines in intelligent construction ecosystems, introduces distinct safety management challenges that necessitate effective emotional resource allocation strategies. This study utilizes job demands-resources theory to investigate how emotional resources are allocated for safety management by examining the relationships among safety practice, psychosocial support, safety participation, and leadership safety behavior.

**Methods:**

A face-to-face questionnaire survey was conducted with front-line construction workers involved in Construction 5.0 projects, yielding 118 valid responses. The data were analyzed using linear regression models and the bootstrap method.

**Results:**

The findings reveal that: (1) psychosocial support positively influences both safety participation and safety practices; (2) safety participation enhances safety practices; (3) safety participation fully mediates the effect of psychosocial support on safety practices; and (4) leadership safety behavior positively moderates the relationship between safety participation and safety practices.

**Conclusion:**

This study extends the job demands-resources framework by illustrating the flow of emotional resources in the context of Construction 5.0, thereby highlighting the principles of multi-level emotional resources in the relationship between emotion and safety. Practically, this framework allows for the evolution of human-centric safety measures in tandem with advanced technology-enabled work environments, while also maintaining psychosocial balance in intelligent construction ecosystems.

## 1 Introduction

Construction 5.0 aims to improve workplace safety, comfort, and ergonomics by combining workers' needs with cutting-edge technologies such as the internet of things (IoT), robotics, and artificial intelligence (AI), while appreciating workers‘ expertise (Ohueri et al., 2024; Wu et al., 2023). This paradigm shifts the industry's focus beyond physical safety to encompass mental wellbeing, emphasizing proactive emotional management in high-stress environments (Hadi et al., 2023; Yang et al., 2023). To align technological advancements with the safety skills of workers and improve workplace conditions, various safety regulations have been established to guide the behavior of construction personnel (Cardoso et al., 2025; Ning et al., 2024). While these regulations are intended to protect workers, they also create additional challenges (Jia et al., 2019). Workers must devote extra time and effort to balance their job duties with compliance, which can lead to increased stress and difficulties in their daily tasks (Jia et al., 2019; Ning et al., 2024). The inherent challenges of Construction 5.0, including dynamic project environments, multifactorial hazards, and escalating quality expectations, further strain workers through intensified workloads and fatigue (Cardoso et al., 2025; Ikudayisi et al., 2023). Fatigue-induced impairments in attention and decision-making, combined with fragmented access to real-time safety resources, amplify risks in volatile operational contexts (Encrenaz et al., 2019; Pooladvand and Hasanzadeh, 2022; Xia et al., 2022b). As a result, construction sites continue to experience a high rate of accidents and injuries despite technological advancements (Hussain et al., 2024; Ning et al., 2024). Compounding these issues, insufficient emotional support exacerbates worker disengagement (Berglund et al., 2024; Ray et al., 2017; Xia et al., 2022b). Stress and burnout, stemming from under-resourced emotional safety programs, often foster resistance to safety regulations (Liu et al., 2025; Xia et al., 2022b). This resistance manifests in counterproductive behaviors, such as feigning compliance with safety protocols (Jia et al., 2019; Xia et al., 2023). Ultimately, these issues undermine effective safety practices and pose a significant threat to the overall success of construction projects (Maali et al., 2024; Xia et al., 2024).

Psychosocial support plays a crucial role in creating a positive emotional environment for construction workers by addressing their emotional needs and fostering a connection between emotions and workplace practices in Construction 5.0 (Berglund et al., 2024; Ikudayisi et al., 2023; Malińska and Bugajska, 2021). By implementing psychosocial support, construction workers can better navigate the challenges they face throughout their careers and adapt to rapid technological shifts (James, 2000; Ohueri et al., 2024). Specifically, psychosocial support can positively influence job characteristics, organizational roles, social dynamics, career development, and overall organizational factors, helping to reduce exhaustion and stress raised in Construction 5.0 (Berglund et al., 2024; Hadi et al., 2023; Pirzadeh et al., 2022; Sobeih et al., 2009). However, psychosocial support alone cannot fully reconcile emotional management with the safety objectives of Construction 5.0 (Arbin et al., 2021; Jiménez Rios et al., 2024; Vieira et al., 2016). Safety participation and leadership safety behavior are also essential in shaping individual emotional responses and the organization's attitude toward safety regulations (Huang et al., 2011; Liang et al., 2021b). On one hand, workers' willingness to engage in additional safety-related behaviors is closely linked to safety participation, which emphasizes individual safety actions (Wei et al., 2016; Zong et al., 2025). On the other hand, construction workers view the organization's commitment to safety and resource allocation as indicators of support reflected in leadership safety behavior (Laurent et al., 2018; Zhang et al., 2016). Despite their theoretical importance, the interplay of psychosocial support, safety participation, and leadership safety behavior remains underexplored in Construction 5.0 projects. A systematic analysis of how these factors interact to shape safety practices is necessary, particularly as construction projects increasingly rely on balancing human wellbeing with technological efficiency.

To fill this gap, the Job Demands-Resources theory is applied to examine how construction workers' emotional wellbeing influences safety practices in Construction 5.0. This framework highlights strategies for optimizing emotional resource allocation to enhance safety management in technology-intensive environments. The authors specifically aim to explore the effects of psychosocial support and safety participation on safety practices, the mediating role of safety participation in the relationship between psychosocial support and safety practices, and the moderating role of leadership safety behavior on the relationship between safety participation and safety practices. This analysis seeks to clarify how psychosocial support, safety participation, and leadership safety behavior shape safety practices in Construction 5.0 settings, linking emotional arousal and reactions to organizational emotional interventions and safety outcomes. This study contributes to the practice of emotional management in Construction 5.0 by highlighting two key recommendations. First, it suggests that managers in temporary organizations should integrate emotional wellbeing initiatives with technological advancements to improve safety outcomes without compromising human-centric values. Second, it emphasizes the need for managers to understand how emotional states affect safety practices in Construction 5.0, ensuring safety practices evolve alongside technological adoption.

## 2 Background and hypotheses

### 2.1 Job demands-resources theory

Job demands-resources (JD-R) theory is used to describe the relationships between burnout in terms of job demands and work engagement in terms of job resources (Lazarova et al., 2010). (Xia et al. 2020) proposed that all job characteristics can be classified into job demands and job resources according to the JD-R theory. Job demands relating to specific contextual factors refer to “those physical, psychological, social, or organizational aspects of the job that necessitate sustained physical and psychological effort and are consequently associated with specific physiological and psychological costs” (Xia et al., 2020). Job resources relating to the enhancement of work engagement are defined as “those physical, psychological, social, or organizational aspects of the job that are functional in achieving work goals, reduce job demands and the associated physiological and psychological costs, or stimulate personal growth, learning, and development” (Cheung et al., 2021; Xia et al., 2020). In a particular context, the interactions between work demands and job resources are assumed to be supported by health impairment and motivational processes in JD-R theory (Yaris et al., 2020). In particular, the health impairment process is designed to elucidate the manner in which job demands deplete employees' energy and result in burnout, whereas the motivational process demonstrates the mechanisms by which job resources promote employees' positive behaviors to achieve relevant goals and facilitate work engagement (Cheung et al., 2021; Conchie et al., 2013; Yaris et al., 2020). In the context of Construction 5.0, workers encounter increased safety challenges that require them to adapt to new technologies and performance expectations. The cognitive and time investments needed to meet these evolving safety protocols surpass those of traditional practices, making it crucial to fulfill safety demands effectively. The ability to meet these safety demands significantly influences the safety practices of construction workers. Psychosocial support, safety participation, and leadership safety behavior can enhance safety-related job resources by providing emotional backing, improving safety perceptions, and addressing organizational issues. Such support can motivate workers to adopt safer practices, increasing their engagement in safety-related tasks and ultimately enhancing safety outcomes in Construction 5.0. Therefore, the motivational processes highlighted in JD-R theory offer a valuable framework for understanding how psychosocial support, safety participation, and leadership safety behavior impact safety practices in this modern construction environment.

### 2.2 Construction workers' safety practice and its role

Safety practice in Construction 5.0 embodies a human-centric approach that proactively integrates advanced technologies with worker behavior to optimize occupational wellbeing (Gambatese John et al., 2017; Osborne et al., 2024). Emerging as an evolution from conventional safety management, this concept transcends mere safety performance evaluation by emphasizing construction workers' real safety behaviors and actual safety outcomes in technology-intensive workspaces (Hwang and Soh, 2013). Organizations invest in tools such as AI-driven training programs, IoT sensors for hazard detection, and ergonomic exoskeletons, while workers adapt to interfaces like augmented reality (AR) safety guides and robotic coordination systems (Han et al., 2020; Wijayarathne et al., 2024). In dynamic Construction 5.0 settings, including hybrid workspaces where humans and machines operate collaboratively, safety outcomes depend not only on resources but also on effective interactions between workers and technology (Liang et al., 2021a; Zhang et al., 2016). For instance, wearable devices that adjust workflows based on fatigue data demonstrate how proactive behaviors and technological tools jointly mitigate risks (Bayram, 2020; Feng et al., 2014). At the organizational level, safety practices involve simulating protocols through digital twin models to predict and prevent hazards (Yan et al., 2025). At the individual level, workers engage with adaptive tools such as smart helmets that provide real-time feedback on posture or equipment use (Ellis and Lee, 2006). Ultimately, more effective safety practices in Construction 5.0 are achieved through workers' exhibition of safer behaviors that generate positive safety outcomes, supported by advanced technological tools.

Safety practices in Construction 5.0 focus on achieving effective safety outcomes through the safe and compliant behaviors of construction workers. It ensures that safety measures are both practical and impactful, helping to reduce accidents in the industry. Various stakeholders (including owners, designers, contractors, and vendors) collaborate to implement these safety measures (Choe et al., 2020). The objective is to enable organizations and workers to effectively utilize safety resources to meet their safety goals (Pritchard et al., 2008). Improvements in safety practices, supported by real-time monitoring and predictive analytics, can significantly lower the risk of project failures and minimize time and cost overruns related to safety management (Hwang and Soh, 2013; Krajangsri and Pongpeng, 2017; Nasirzadeh and Nojedehi, 2013). While psychosocial factors, safety climate, and organizational behaviors remain critical in Construction 5.0, they are increasingly influenced by technological interfaces (Mitropoulos and Memarian, 2012). Issues such as absenteeism and presenteeism are linked to psychosocial factors like social support and the physical work environment, which can negatively impact safety outcomes (Abdullah et al., 2015; Bayram, 2020; Johns, 2010). The safety climate in Construction 5.0 is shaped by shared perceptions of safety policies and practices, as well as trust in automated systems and the transparency of AI-generated safety protocols (Ohueri et al., 2024; Xia et al., 2021). This evolving climate indirectly influences workers' safety behaviors, affecting their compliance with hybrid workflows that combine human decisions with machine recommendations (Barling et al., 2002; Bonham et al., 2017; Cooper and Phillips, 2004). Organizational safety behaviors in Construction 5.0 focus on upskilling workers to safely interact with automated machines, fostering psychological safety in data-driven decision-making, and encouraging proactive reporting of system vulnerabilities (Wang et al., 2021; Wijayarathne et al., 2024). These initiatives create a secure human-technology ecosystem and facilitate effective safety practices through adaptive governance frameworks (Ford and Tetrick, 2008).

### 2.3 Psychosocial support and its effect on safety practice

Psychosocial support is defined as a form of support characterized by friendship and caring that extends beyond work (Higgins et al., 2010). In Construction 5.0 environments where human-robot collaboration becomes increasingly common, psychosocial support emphasizes the establishment of emotional support and trust through hybrid human-digital interfaces, which can provide support in the areas of care and friendship while maintaining technological synergy (James, 2000; Zhuang et al., 2013). Emotional support in smart construction sites requires demonstrating courtesy and regard for construction workers through both interpersonal interactions and digitally-mediated communication channels, which reduces technostress levels in technology-intensive work environments (Tews et al., 2020). Contemporary trust-building extends beyond human relationships to encompass triadic interactions among workers, collaborative robots, and AI systems, facilitated by algorithmically transparent processes that enhance human-machine team cohesion (Chua et al., 2008). Mentoring functions and psychosocial factors remain crucial in Construction 5.0 contexts. Individuals receiving psychosocial support through AR training systems exhibit higher professional optimism, enhancing their resilience in adapting to digital-physical work ecosystems while reducing automation-related stress (Qin and Bulbul, 2023). Their career outcomes now include digital skill satisfaction and emotional investment in sustainable construction practices (Higgins et al., 2010). Advanced implementations integrate IoT-embedded social support systems that perform continuous behavioral monitoring, enabling predictive wellbeing interventions to reduce burnout and real-time hazard detection to prevent accidents (Huang et al., 2019; Sobeih et al., 2009). While traditional demographic variables remain relevant, Construction 5.0 introduces digital literacy as a critical demographic determinant influencing psychosocial support accessibility (James, 2000). The reconceptualized psychosocial perspective advocates hybrid supervisory models that synthesize AI-powered performance analytics with human-centered leadership principles (Callari et al., 2024). These models leverage collaborative digital twins as immersive communication platforms to optimize team dynamics, effectively bridging technological capabilities with psychosocial needs (Du et al., 2020; Ingelgård et al., 1996).

Safety management in Construction 5.0 establishes multidimensional job requirements that integrate intelligent monitoring systems with human-centered safety protocols (Chauhan et al., 2024; Chen and Chan Isabelle, 2024; Ohueri et al., 2024). Construction workers' safety practices emerge as the critical output of their safety input, mediated through the expenditure of safety-related resources (Bayram, 2020; Dai et al., 2009). In this context, psychosocial support plays a crucial role in technology-intensive workspace by enhancing workers' social connections and self-efficacy—key emotional resources needed for effective human-machine collaboration Dollard et al., 2000; Higgins et al., 2010). According to JD-R theory, when construction workers receive adequate psychosocial support, they are better equipped to manage the cognitive demands of hybrid physical-digital safety operations (Xia et al., 2023). This support helps maintain their cognitive engagement with safety objectives, leading to improved compliance with adaptive safety regulations (Higgins et al., 2010; James, 2000; Lee et al., 2020). Therefore, psychosocial support serves as a vital resource within Construction 5.0, compensating for the psychological effort needed to sustain safety vigilance in technology-driven environments (Ohueri et al., 2024; Pirzadeh et al., 2022). It also promotes positive safety outcomes through enhanced peer-to-peer knowledge sharing (Higgins et al., 2010; Lee et al., 2020). This mechanism suggests that psychosocial support interventions can significantly enhance the effectiveness of safety investments in Construction 5.0 and foster sustained safety behaviors and practices that exceed basic compliance. Thus, the first hypothesis is proposed as follows:

**Hypothesis 1 (H1)**: psychosocial support has a positive effect on safety practice.

### 2.4 Safety participation and its mediating role between psychosocial support and safety practice

Safety participation is defined as behaviors that do not directly contribute to an individual's personal safety but that do help to develop an environment that supports safety (Xia et al., 2018a). In the context of Construction 5.0, this concept expands to include collaborative behaviors between humans and technology, where workers engage with digital tools such as digital twins and safety systems (Wijayarathne et al., 2024). Modern forms of safety participation involve activities like AR-enhanced safety drills, collaborative problem-solving on digital safety platforms, and proactive reporting of issues within building information modeling (BIM) systems (Hussain et al., 2024; Shekargoftar et al., 2022). As a critical element of employee safety performance behaviors, safety participation in Construction 5.0 not only sustains traditional safety climate but also optimizes human-cyber-physical system interoperability in smart construction ecosystems (Ohueri et al., 2024). This integration allows for the identification of system dysfunctions and promotes safe actions in uncertain situations (Martínez-Córcoles et al., 2012). Prior studies demonstrate the competencies required in Construction 5.0, such as digital literacy for managing safety analytics and cognitive flexibility for working alongside robots, complement traditional predictors like safety knowledge and motivation observed in manufacturing and healthcare sectors (Ohueri et al., 2024; Yu et al., 2021). Moreover, workers' trust in automation systems plays a crucial role in moderating safety participation (Strazzullo, 2024). However, the optimism associated with adopting new technologies in Construction 5.0 may inadvertently reduce safety participation if workers become overly reliant on automated safety measures (He et al., 2019).

In Construction 5.0, the fulfillment of safety-related job demands for workers relies on the combined support of emotional resources and technology-enhanced contextual resources. Drawing on the JD-R theory, safety participation can endow construction workers with contextual safety-related resources by cultivating a safety climate (Laurent et al., 2018). First, in collaborative human-robot work scenarios, the contextual resources, such as recognition mechanisms through real-time digital feedback systems, can assist workers in recognizing the safety behaviors that are encouraged by fostering positive safety perceptions (Laurent et al., 2018). This will eliminate the extraneous behaviors of construction workers and encourage them to concentrate more on safety engagement. Consequently, the waste of safety-related resources is reduced, which in turn generates additional opportunities to obtain additional safety practices. Second, psychosocial support, as a human-centric emotional resource, can boost the sense of team identification and job security among workers in Construction 5.0 Jiménez Rios et al., 2024; Laurent et al., 2018). This, in turn, motivates them to invest additional resources, such as those accessible through virtual reality (VR) training modules and real-time communication tools, in safety work, thus increasing their safety participation (Dollard et al., 2000; Guo et al., 2019). A high level of psychosocial support has been associated with enhanced safety participation (Guo et al., 2019; Higgins et al., 2010). Third, H1 posits that in Construction 5.0, psychosocial support can enhance safety practices by improving workers' utilization efficiency of psychosocial resources. Through safety participation, with the assistance of IoT and sensor technology, a psychosocial safety climate can be established. This climate encourages workers to engage in more safety activities, allowing for the dynamic allocation of safety resources according to their job responsibilities (Dollard et al., 2012). This demonstrates that safety participation in Construction 5.0 not only replenishes contextual resources through digital workflow integration but also ensures the efficient utilization of psychosocial resources for better safety practices. Based on the above-mentioned discussion, three hypotheses are proposed:

**Hypothesis 2 (H2)**: safety participation has a positive effect on safety practice.
**Hypothesis 3 (H3)**: psychosocial support has a positive effect on safety participation.
**Hypothesis 4 (H4)**: safety participation mediates the effect of psychosocial support on safety practice.

### 2.5 Leadership safety behavior and its moderating role

Leadership safety behavior refers to strategic organizational actions that foster employees' safety consciousness during task execution through structured managerial interventions (Cheng et al., 2020; Zhang et al., 2017). In the context of Construction 5.0, which integrates advanced digital technologies and automation, these actions include developing IoT-integrated safety protocols, implementing AI-driven risk assessment systems, conducting VR-enhanced safety training, and establishing real-time safety communication channels through digital collaboration platforms (Zhang et al., 2017). Within smart construction environments, leadership safety behavior not only demonstrates organizational competence in safety management but also reflects leaders' commitment to worker welfare through technology-mediated protective measures (Li et al., 2018; Tabassi et al., 2017). Leaders in Construction 5.0 face the challenge of managing human-robot collaboration and the deployment of IoT devices, both of which introduce new safety complexities (Ohueri et al., 2024; Zafar et al., 2024). Effective leadership safety behavior is essential for improving safety performance by ensuring that employees receive proper training in new technologies and that safety protocols are regularly updated to address emerging risks (Zhang et al., 2017). Additionally, this behavior fosters greater safety awareness among employees and encourages the correction of unsafe practices, particularly in the rapidly evolving environment of Construction 5.0 (Cheng et al., 2020). As a type of leadership safety behavior, leadership safety behavior is influenced by both internal and external factors (Zhang et al., 2017). Internally, leaders' risk preferences are shaped by individual safety attitudes, differences, motivations, and safety skills, which can influence their leadership styles and subsequently their leadership safety behavior (Wang and Zhao, 2019; Zhang et al., 2017). Externally, factors such as social capital, safety policies, and suppliers' requirements, which determine situational factors, can affect leadership safety behavior by shaping conditions outside the organizations (Courtright et al., 2014; Zhang et al., 2017). In Construction 5.0, these factors also include the integration of new technologies, regulatory compliance related to digital infrastructure, and the need for continuous adaptation to technological advancements (Ohueri et al., 2024; Shirwa et al., 2025).

As posited in H2, safety participation empowers construction workers to discern contextually appropriate safety behaviors within Construction 5.0 environments, effectively minimizing non-value-adding actions and resource misallocation while generating enhanced safety practice opportunities. Functioning as a techno-managerial interface, leadership safety behavior amplifies this mechanism through dynamic provisioning IoT-integrated safety protocols, AI-driven risk assessment systems, VR-enhanced safety training, real-time safety communication channels, and so forth (Hoffmeister et al., 2014; Tummers and Bakker, 2021). This integration not only solidifies an organization's commitment to safety but also helps workers develop a contextual understanding of safety objectives through immersive digital tools, thereby increasing the effectiveness of safety practices (McConville et al., 2016; Zhang et al., 2017). In line with the JD-R theory, safety participation (as psychological resource) and leadership safety behavior (as organizational resource) synergistically address the job demands of safety practice in Construction 5.0 (Xia et al., 2020). In this context, leadership safety behavior can be seen as an environmental factor that creates favorable conditions for safety participation and practices (Graham et al., 2020). Strong leadership safety behavior provides additional organizational resources that enhance safety participation by establishing various safety contexts through technology-driven interventions (Griffin and Hu, 2013; Xia et al., 2022a). This encourages workers to focus on safety activities and improves overall safety practices. Conversely, weak leadership safety behavior leads to fragmented digital safety networks and underutilized predictive maintenance systems (Griffin and Hu, 2013; Xia et al., 2018b). This lack of technological engagement reflects an organization's ambivalence toward safety, which can diminish workers' psychological commitment to safety activities and hinder their ability to meet the evolving safety demands of smart construction environments (Griffin and Hu, 2013; Neal and Griffin, 2006). Ultimately, leadership's ability to integrate digital and human safety resources critically determines the efficacy of safety participation-practice conversion. Thus, the fifth hypothesis is proposed as follows:

**Hypothesis 5 (H5)**: leadership safety behavior moderates the relationship between safety participation and safety practice. Specifically, the positive impact of safety participation on safety practice is stronger when leadership safety behavior is high than when it is low.

**Figure 1:**
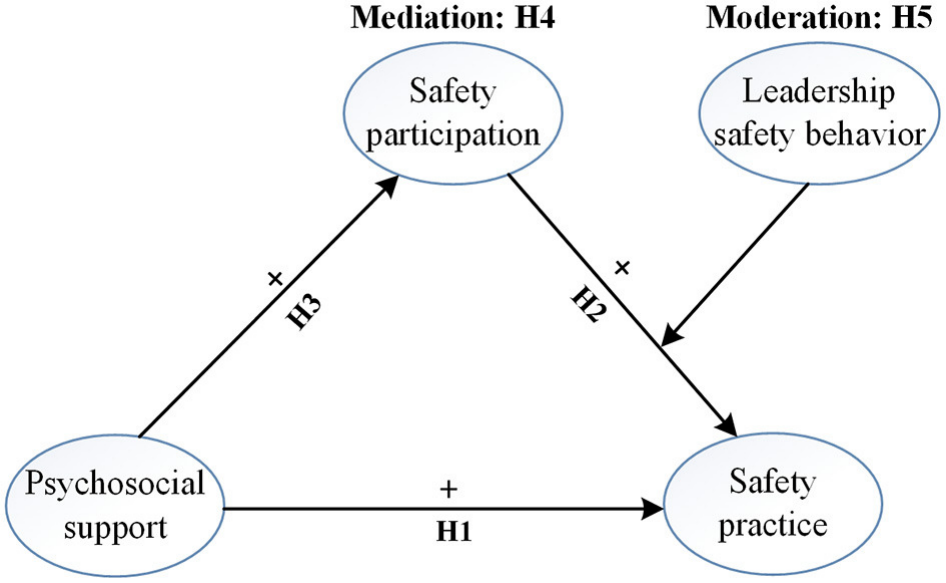
Conceptual model.

Figure 1 illustrates the conceptual model in accordance with H1, H2, H3, H4 and H5.

## 3 Methodology

### 3.1 Questionnaire design and survey

A questionnaire survey can not only collect massive data from target groups but also explore general views of construction workers on variables (Mohamed, 2002; Ng et al., 2005). Questionnaire surveys are widely applied to measure and analyze relationships between variables in construction projects (Hwang et al., 2018; Romm, 2013). This study aims to test the hypotheses specified in Figure 1. Therefore, a face-to-face questionnaire survey was conducted from April to August 2023 in order to test the hypothesized relationships between psychosocial support, safety participation, safety practice, and leadership safety behavior in Construction 5.0.

The questionnaires were initially prepared in English but were translated into Chinese to accommodate the workers' language needs, followed by back-translation into English for accuracy. The questionnaires consisted of two sections: the first focused on background information, while the second examined four key areas: psychosocial support, safety participation, safety practices, and leadership safety behavior. The survey concentrated on projects exemplifying the attributes of Construction 5.0, encompassing intelligent construction and smart construction sites. Before distributing the formal questionnaire, a pilot study was conducted in Nanjing to gather feedback from workers on the initial version. Their insights led to improvements in the clarity and readability of certain items, resulting in the finalized questionnaire. To ensure data reliability, a purposive sampling technique was employed (Ahmed et al., 2022). Additionally, considering that factors such as gender, experience, and education can influence psychosocial support, safety participation, safety practice, and leadership safety behavior in Construction 5.0 (O'brien et al., 2010; Vithanage et al., 2022), specific criteria were established for respondents: (1) they must be front-line workers in Construction 5.0, like intelligent or smart construction sites, (2) they should use intelligent tools like welding, plastering, and spraying robots and so forth, (3) both skilled and unskilled female workers were included, and (4) they should have prior experience with accidents. Consequently, locations meeting these criteria were selected to conduct the survey for this study on various Chinese construction projects.

Construction workers were able to complete the questionnaire in the project meeting room during their meal breaks, with significant support from the project managers. Participants were instructed to answer the questions based on their current or past project experiences. To encourage participation, each respondent received a small gift, consisting of a toothbrush and a towel, for completing the questionnaire. In total, 200 questionnaires were distributed, resulting in 118 valid responses for data analysis. The sample reflected a diverse demographic, encompassing various factors such as gender, age, occupation, work experience, education level, daily income, and project location (see Table 1).

**Table 1:** Information of the surveyed workers.

**Information**	**Categories**	**Number**	**Percentage %**	**Information**	**Categories**	**Number**	**Percentage %**
Gender	Male	81	68.64	Daily income (CNY)	< 200	42	35.59
	Female	37	31.36		200-300	32	27.12
Age (years)	16-30	40	33.89		300-350	27	22.88
	30-40	53	44.91		>350	17	14.41
	40-50	21	17.80	Education level	Junior high school or below	29	24.58
	>50	4	3.4		High school	16	13.56
Occupation	Carpenter	7	5.93		Associate's degree	23	19.49
	Steel bender	15	12.71		Bachelor's degree	38	32.20
	Crane driver	24	20.34		Master's degree or higher	12	10.17
	General worker	71	60.17	Project location	East China	28	23.73
	Others	1	0.85		North China	7	5.93
Working experience (years)	< 2	14	11.86		Central China	5	4.24
	2-5	33	27.97		Southern China	11	9.32
	5-10	32	27.12		Southwest China	4	3.39
	10-15	26	22.03		Northwestern China	62	52.54
	>15	13	11.02		Northeastern China	1	0.85

### 3.2 Measures

#### 3.2.1 Safety practice

Safety practice in Construction 5.0 indicators include the following: enhanced safety competency through reasonable collaborative interfaces, reduced safety preparation time enabled by digital risk assessment checklists, improved worker health status through monitored systems, optimized safety equipment adjustment time through environmental adaptive approach, increased intrinsic safety awareness through VR enhanced situational training, and decreased safety-related complaints through scenario simulations (Bayram, 2020; Hasanzadeh and De La Garza, 2020; Johari and Jha, 2020). One sample item was “To what extent does VR-enhanced situational training improve your safety awareness on this construction project?” Participants rated their responses using a 5-point Likert scale, where 1 indicated “completely unhelpful” and 5 indicated “very helpful”.

#### 3.2.2 Psychosocial support

The assessment of psychosocial support in Construction 5.0 environments incorporates five human-technology integration indicators, measured through: technology-integrated direction and guidance, digital-era role modeling, timely digital support during periods of stress, affirmation of innovative construction ideas, and smart-connected friendly relationships among colleagues, as adapted from (Methot and Cole 2021), (Parker et al. 2019), (Thomas 1990), and (Trau 2015). The measurement tool includes context-specific questions, such as “To what extent do digital collaboration platforms provide timely guidance on equipment optimization for this construction project?” Responses were rated using a 5-point Likert scale, where 1 indicated “no noticeable effect” and 5 indicated “highly significant effect”.

#### 3.2.3 Safety participation

Four indicators were developed to measure safety participation in Construction 5.0, drawing from the research of (Dearmond et al. 2011), (Kalteh et al. 2021), and (Neal and Griffin 2006). The four indicators were: (1) willingness to make additional efforts leveraging digital tools to improve the safety of the construction site, (2) willingness to engage in non-mandatory, technology-integrated safety-orientated training, (3) willingness to mentor other workers using digital platforms to ensure that they perform their tasks safely, (4) willingness to attempt to modify the job's execution by integrating smart technologies to enhance its safety. The measurement instrument incorporated context-specific items such as “To what extent are you willing to make additional efforts leveraging digital tools to improve the safety of the construction site?” All items were evaluated using a 5-point Likert scale (1 = not at all willing to 5 = extremely willing).

#### 3.2.4 Leadership safety behavior

To assess leadership safety behavior in Construction 5.0, three key indicators proposed by (Zhang et al. 2017) were utilized. These indicators are: (1) the establishment of a digital-enabled safety responsibility system by leaders, (2) encouragement for workers to submit safety recommendations through digital channels, and (3) the implementation of a data-driven safety incentive system that includes rewards and recognition. One example of a survey item is, “How well-developed is the digital-enabled safety responsibility system established by your leaders for this construction project?” Participants evaluated each item using a five-point Likert scale, where 1 indicated “not developed at all” and 5 indicated “very well developed”.

### 3.3 Analytic strategy

Initially, Cronbach's alpha coefficients were calculated for safety practices, psychosocial support, safety participation, and leadership safety behavior measures using SPSS 23.0 to assess their reliability before testing the hypotheses. Next, confirmatory factor analyses (CFAs) were conducted with Amos 21.0 to evaluate the convergence and discriminant validity of the four constructs. Following this, regression models were developed to analyze the proposed relationships (H1, H2, and H3). Finally, the bootstrapping method, involving 5,000 resampling iterations, was used to assess the mediating and moderating effects outlined in hypotheses H4 and H5.

The Bootstrap method enhances the accuracy and power of mediation and moderation estimates by constructing confidence intervals (CIs) (Russell and Dean, 2000; Vance et al., 2015). This method establishes a 95% bias-corrected CI by resampling 5,000 times from the original sample, following the three-step process outlined by (Baron and Kenny 1986). Notably, this approach does not rely on the assumption of normally distributed compound coefficients (Edwards and Lambert, 2007; Vance et al., 2015; Xia et al., 2022a). A significant mediating effect is indicated when the CI does not include zero (Lau and Cheung, 2012). To test for moderating effects, three standardized predictors are required: the independent variable (e.g., safety participation), the moderator (e.g., leadership safety behavior), and their interaction (e.g., safety participation × leadership safety behavior) (Cohen et al., 2003; Preacher et al., 2007). A moderation effect is confirmed if the interaction term is statistically significant (Xia et al., 2022a).

## 4 Results

### 4.1 Reliability testing

Reliability testing is applied to examine the internal consistency of a questionnaire survey, and the degree of the internal consistency can be expressed by the values of Cronbach's alpha (Xia et al., 2022a). The value is considered adequate when the alpha value exceeds 0.70 (Zhang et al., 2020). The Cronbach's Alphas of the current sample are 0.895, 0.892, 0.917, and 0.913 for the safety practice, psychosocial support, safety participation, and leadership safety behavior, respectively.

### 4.2 Validity testing

First, series of CFAs were conducted in AMOS 21 to access the uncorrelated degree of the four constructs (e.g., safety practice, psychosocial support, safety participation, and leadership safety behavior). The results show that the four-construct model had a reasonable fit to the data [χ^2^ = 212.66, *p* < 0.01; degree of freedom (*Df* ) = 102, comparative fit index (CFI) = 0.952, Tucker-Lewis index (TLI) = 0.928; standardized root mean square residual (SRMR) = 0.047; and root mean square error of approximation (RMSEA) = 0.096], and outperformed the following models: the two-construct model that collapses psychosocial support, safety participation, and leadership safety behavior (χ^2^ = 670.66, *p* < 0.01; *Df* = 134, CFI = 0.768, TLI = 0.736; SRMR = 0.078; RMSEA = 0.185); three-construct model that collapses psychosocial support and safety participation (χ^2^ = 647.13, *p* < 0.01; *Df* = 132, CFI = 0.778, TLI = 0.742; SRMR = 0.075; RMSEA = 0.183); three-construct model that collapses safety participation and leadership safety behavior (χ^2^ = 632.49, *p* < 0.01; *Df* = 132, CFI = 0.784, TLI = 0.750; SRMR = 0.077; RMSEA = 0.180); three-construct model that collapses leadership safety behavior and psychosocial support (χ^2^ = 644.348, *p* < 0.01; *Df* = 132, CFI = 0.779, TLI = 0.744; SRMR = 0.076; RMSEA = 0.182); and one-construct model that collapses all of the four construct (χ^2^ = 723.822, *p* < 0.01; *Df* = 135, CFI = 0.746, TLI = 0.712; SRMR = 0.068; RMSEA = 0.193) (Hu and Bentler, 1999). The four-construct model performs better than other models, demonstrating its superiority in result analysis.

Second, averaging the variance extracted (AVE) illustrated their convergent validity by gauging the extent of their overlap. Convergent validity is established when the values of all the variables are greater than 0.50 (Garbarino and Johnson, 1999). The AVEs for safety practice, psychosocial support, safety participation, and leadership safety behavior are 0.743, 0.596, 0.653, and 0.752 respectively, indicating convergent validity.

### 4.3 Hypothesis Testing

#### 4.3.1 Main effects

The main effects proposed in Hypotheses 1, 2, and 3 were tested using three regression models in SPSS 23, as mentioned in the section 3.3. Model 1 examined the influence of psychosocial support on safety practice, Model 2 examined the impact of safety participation on safety practice, while Model 3 examined the impact of psychosocial support on safety participation. The results of the three models are displayed in Table 2.

**Table 2:** Results of main effects.

**Predicted paths**	**Model 1 (Hypothesis 1)**		**Model 2 (Hypothesis 2)**		**Model 3 (Hypothesis 3)**	
	**Estimate**	**S.E**.	**Estimate**	**S.E**.	**Estimate**	**S.E**.
Psychosocial support → Safety practice	0.695^***^	0.063				
Safety participation → Safety practice			0.903^***^	0.053		
Psychosocial support → Safety participation					0.735^***^	0.050

^***^ Indicates P is estimated below the 0.001 significance level.

Estimate is unstandardized regression coefficient, and S.E. is the standard error of the estimate.

Hypothesis 1 and Hypothesis 2 are supported by Models 1 and 2, which both demonstrate significant and positive relationships between psychosocial support and safety participation and safety practice. Additionally, the results of Model 3 psychosocial support generally support H3 since they are positively correlated with safety participation.

#### 4.3.2 Mediating effects

Using Hayes' PROCESS macro Model 4 for SPSS, the bootstrapping method was employed to evaluate safety participation's mediating role in the relationship between psychosocial support and safety practice (Hayes, 2017). According to (Dobrucali and Özkan 2021), a bootstrapping of 5,000 samples was performed to test H4. The results of the hypothesis testing are presented in Table 3.

**Table 3:** Results of mediating effects.

**Psychosocial support → Safety**	**Estimate**	**S.E**.	**CI**	
**participation → Safety practice**			**LLCI**	**ULCI**
Total effect	0.695^***^	0.084	0.529	0.860
Direct effect	0.089	0.145	−0.199	0.377
Indirect effect	0.824^***^	0.109	0.402	0.826

^**^ Indicates the significance level of P is below 0.01.

^***^ Indicates the significance level of P is below 0.001.

Estimate is unstandardized regression coefficient, S.E. is the standard error of the estimate, CI is confidence interval, LLCI is the lower bound of CI, and ULCI is the upper bound of the CI.

The results presented in Table 3 show the mediating effects hypothesized in H4 were validated. The indirect effect of psychosocial support via safety participation on safety practice is positive and significant, as the 95% bias-corrected CI ([0.402, 0.826]) excludes zero. Furthermore, given that the CI for the total effects of psychosocial support on safety practice exclude zero, these effects are positive and significant. In contrast, the direct effects of psychosocial support on safety practice, which contains zero in the corresponding CI, are not significant. This indicates that safety participation fully mediates the relationship between psychosocial support and safety practice.

#### 4.3.3 Moderating effects

The moderating effect of leadership safety behavior in the relationship between safety participation and safety practice was tested by bootstrapping method (5,000 resamples) through Model 1 of Hayes' PROCESS macro for SPSS (Hayes, 2017). Table 4 shows the results of the hypothesis testing.

**Table 4:** Results of moderating effects.

**Predictor**	**Estimate**	**S.E**.	**CI**	
			**LLCI**	**ULCI**
Leadership safety behavior	0.474^*^	0.197	0.084	0.865
Safety participation	1.349^***^	0.172	1.009	1.689
Safety participation × Leadership safety behavior	−0.228^**^	0.075	−0.376	−0.080

^*^ Indicates the significance level of P is below 0.05. ^***^ Indicates the significance level of P is below 0.001. ^**^ Indicates the significance level of P is below 0.01. *Estimate* is unstandardized regression coefficient, *S.E*. is the standard error of the estimate, *CI* is confidence interval, *LLCI* is the lower bound of *CI*, and *ULCI* is the upper bound of the *CI*.

**Figure 2:**
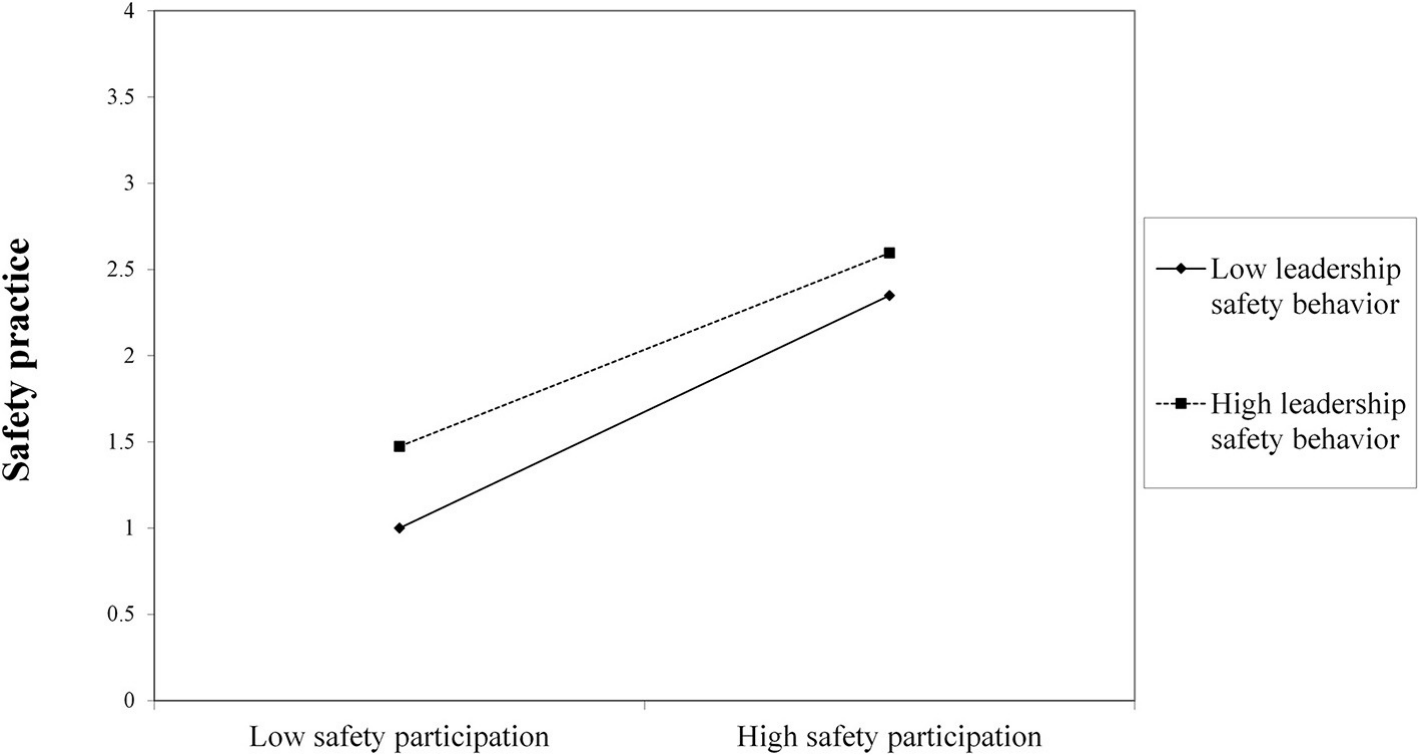
Moderating effect of Leadership safety behavior on the relationship between Safety participation and Safety practice.

According to Table 4, the interaction effect of safety participation with leadership safety behavior (Estimate = −0.228, S.E. = 0.075, *P* < 0.01) on safety practice is statistically significant and positive. This indicates that the link between safety participation and safety practice is positively moderated by the leadership safety behavior. In order to evaluate the impact of the moderator on the relationship between safety participation and safety practice, a basic slope was constructed in Figure 2 using the methods described by (Aiken et al. 1991).

The finding that safety participation exerts a greater positive influence on safety practice when leadership safety behavior is high further supports Hypothesis 5, as illustrated in Figure 2. The extent of safety participation's impact on safety practice can be enhanced by leadership safety behavior.

### 4.4 *Post-hoc* power analysis

**Figure 3:**
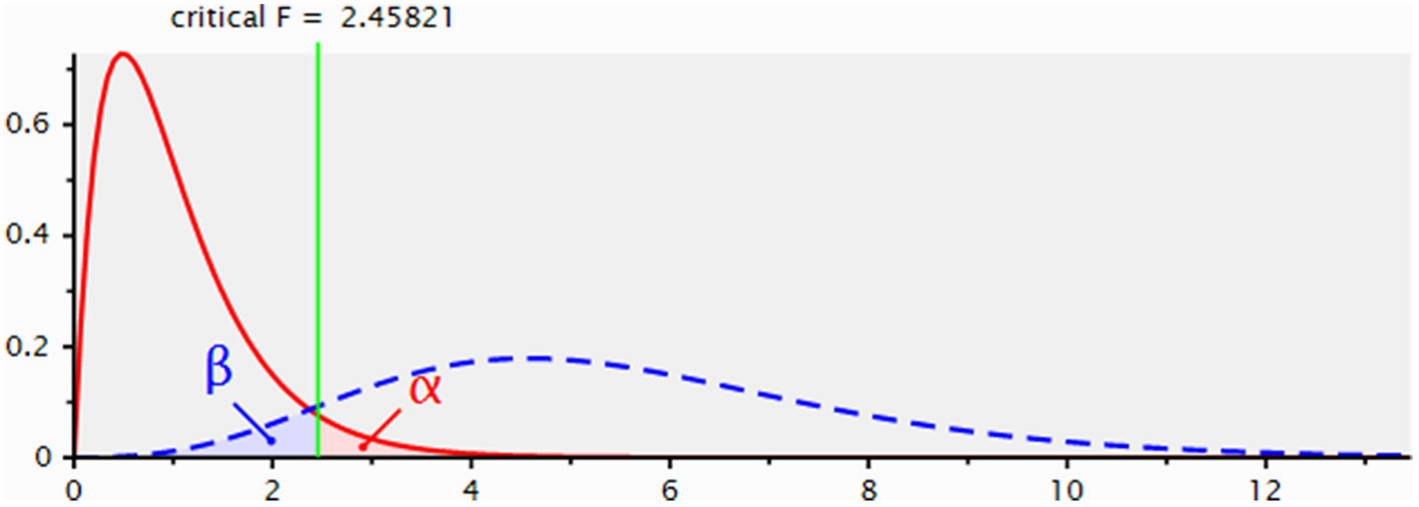
The outcome of *post-hoc* power analysis.

G^*^ Power software was utilized to perform a post-hoc power analysis to assess the robustness of the study's findings. An effect size *f*^2^ of 0.15, a significance level α of 0.05, and a total sample size of 118 are provided as inputs, yielding a critical F of 2.458 and a power of 0.928. Figure 3 displays the results of the post-hoc power analysis.

(Cohen 1988) posits that an effect size *f*^2^ of 0.15 is indicative of a medium effect. Furthermore, a statistical power of 0.928 surpasses the commonly accepted minimum threshold of 0.8 for adequate power.

## 5 Discussion

### 5.1 Positive impact of psychosocial support on safety participation and safety practice

Our study provides novel insights into how psychosocial support enhances safety practices within the dynamic human-cyber collaboration framework of Construction 5.0. The statistically significant result (*P* < 0.001) supporting H1 reveals that psychosocial interventions specifically counteract the emotional resource depletion caused by intelligent system integration defining feature of Construction 5.0 environments. This demonstrates that replenishing workers' emotional reserves through multi-source social support (colleagues, relatives, natives) enables effective safety behavior implementation despite the cognitive demands of human-machine teaming and real-time data processing. The findings establish an emotional equilibrium mechanism that resolves the tension between safety assignment complexity (heightened by cyber-physical systems) and safety practice sustainability-addressing a critical gap in occupational health research within Construction 5.0 contexts. Unlike previous psychosocial studies focused on work-family balance or youth adaptation (Bates et al., 2019; Xia et al., 2018b), our age-diverse sample demonstrates how relational support networks mitigate technology-mediated stressors unique to Construction 5.0, while maintaining alignment with lean management principles through optimized safety resource utilization.

Our findings reveal that psychosocial support's efficacy in enhancing safety participation is fundamentally mediated through human-centric interfaces characteristic of Construction 5.0 ecosystems. The statistically robust relationship (*p* < 0.001) demonstrates that in environments featuring collaborative robotics and digital twin technologies, workers' engagement in safety practices relies on psychosocial mechanisms that address the challenges of human-technology collaboration. Specifically, this includes the need for greater job security in automated workflows and a stronger sense of team identity within hybrid human-robot teams. This evidence builds on the stress-buffering model proposed by (Wang et al. 2018) by integrating it with key elements of Construction 5.0: (1) real-time feedback for proactive interventions, (2) user-friendly interfaces for social support, and (3) a strengthened professional identity within dynamic teams. Crucially, our operationalization of Liu et al.'s (2020) psychological safety construct now incorporates the cyber-physical dimensions of Construction 5.0. Workers' sense of belonging is fostered by both human networks and their confident interactions with collaborative robots. Moreover, safety participation now encompasses not only physical precautions but also active involvement with predictive safety analytics platforms. This dual reinforcement mechanism, where intelligent systems enhance human psychosocial support and vice versa, establishes a new paradigm for safety management in cognitively demanding and technology-rich work environments.

### 5.2 Positive impact of safety participation on safety practice and its mediating role

The novel contribution of this study lies in revealing how safety participation operates as a catalytic converter within Construction 5.0's human-cyber-physical systems. Our findings demonstrate that H2 was statistically significant at the 0.001 level (*P* < 0.001), establishing that safety practice improvements in smart construction environments fundamentally depend on safety participation enhancement. This mechanism manifests through two distinct pathways aligned with Construction 5.0's analytics capabilities: (1) the optimized use of technology-driven safety resources, such as risk prediction and behavioral monitoring, and (2) improved identification and elimination of unsafe activities through collaborative workflows between humans and technology. These findings build on the work of (Laurent et al. 2018), which emphasizes the importance of organizational safety frameworks, by illustrating how worker participation actively engages human-cyber-physical safety systems. This engagement is crucial in Construction 5.0, where human decisions interact with autonomous equipment. Such operational clarity facilitates lean safety management by allowing for dynamic adjustments in behavior-practice trade-offs, particularly beneficial in projects that employ modular construction and sensor technologies. Furthermore, these findings offer computational validation for theoretical proposition of (Bayram 2020). They show that collaborative safety participation, as opposed to simple compliance, is a key driver of improvements in safety practices within technology-intensive work environments. This study clarifies previous uncertainties by providing empirical evidence from smart construction contexts, underscoring the critical role of active engagement in safety processes.

This study presents new evidence that, within the collaborative context of Construction 5.0, safety participation fully mediates the relationship between psychosocial support and safety practices (H4). This finding indicates that the influence of psychosocial support on safety behaviors is entirely channeled through workers' proactive engagement in safety activities. This engagement is crucial in environments characterized by real-time data sharing, advanced technology-driven risk prediction, and human-machine collaboration. Specifically, safety participation enables workers to effectively respond to the dynamic safety demands of Construction 5.0 by directing their emotional resources toward behaviors encouraged by a supportive safety climate. This discovery builds on previous research by (Kocatepe and Parlak 2021) and (Bunner et al. 2018), which examined accident prevention frameworks in smart construction ecosystems. It illustrates how safety participation shapes workers' understanding of safety protocols amid rapid technological changes. Additionally, it highlights how the role of safety participation expands to connect psychosocial factors with technological safety systems, a dimension that has been overlooked in business mediation models by (Yaris et al. 2020). Our empirical validation of this mediation pathway within the fluid organizational structures of Construction 5.0 provides a theoretical foundation for managing safety in temporary smart worksites, where human adaptability interacts with automated systems.

### 5.3 Moderating role of leadership safety behavior

This study reveals novel empirical insights into how leadership safety behavior operates as a techno-managerial catalyst in Construction 5.0 environments. The findings confirm H5, demonstrating that leadership safety behavior positively moderates the relationship between safety participation and safety practice. In smart construction ecosystems, leadership safety behavior amplifies safety participation's efficacy through dynamic provisioning of AI-driven risk analytics, VR-enhanced training modules, and real-time safety communication via digital platforms. This technological mediation enables leaders to systematically reconfigure situational safety factors while embedding organizational safety values into workers' cognitive schemas through immersive digital interfaces. The results advance prior research by (Chu et al. 2021) and (Guo et al. 2019) on organizational safety support by delineating how Construction 5.0's digital infrastructure transforms leadership safety behavior into both a situational shaper (via IoT-integrated interventions) and a psychological anchor (through technology-mediated safety narratives). This dual mechanism strengthens the JD-R theoretical framework by showing how leadership safety behavior, as an organizational resource, interacts with safety participation to address emerging techno-physical job demands. Furthermore, the study extends spiritual leadership model proposed by (Liu et al. 2023), highlighting how digitally enhanced leadership safety behavior reshapes safety-related social norms within the construction workforce. It emphasizes that in Construction 5.0, leaders must effectively coordinate safety data, innovative training, and communication channels to prevent fragmented digital safety systems. This coordination is essential to ensure that workers' psychological safety aligns with evolving workplace demands.

## 6 Theoretical implication

**Figure 4:**
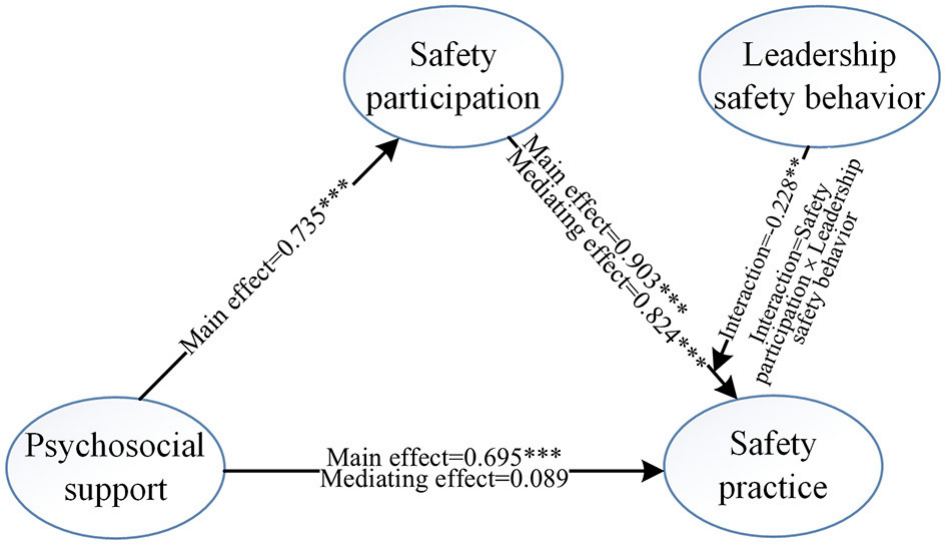
The results of Bootstrap.

This study utilizes JD-R theory to investigate the connections between psychosocial support, safety participation, leadership safety behaviors, and safety practices in Construction 5.0. As illustrated in Figure 4, the results demonstrate how emotional support influences safety practices. These findings are particularly beneficial for temporary project teams, as they help develop adaptive safety mechanisms that respond to fluctuating emotional conditions at worksites. Unlike the effort-reward imbalance (ERI) model, which focuses on perceived structural imbalances within organizations (Vilser et al., 2024), this study emphasizes the internal motivational pathways that drive safety practices among construction workers in the context of Construction 5.0. It underscores the reciprocal relationship between individual cognitive differences and the demands-resources framework. By clarifying the mediating role of safety participation, the study reveals the mechanisms that underpin safety motivation among construction workers, thus enriching the ERI model's perspective on the positive and non-material drivers of safety motivation in high-risk environments. Additionally, this research clarifies the pathway from psychosocial support to safety practices, validating how the three essential psychological needs (i.e. autonomy, competence and relatedness) identified in self-determination theory (SDT) contribute to the internalization of safety behaviors (Luo et al., 2022). By exploring the moderating effect of leadership safety behavior, the study further illustrates how fulfilling these basic psychological needs enhances the effectiveness of motivation transformation. These findings not only broaden the application of converting external resources into motivation within technology-driven environments but also provide a practical framework for sustaining safety motivation in high-risk contexts.

## 7 Practical implication

This study outlines two practical approaches for implementing psychosocial support within the human-digital ecosystems of Construction 5.0, where advanced technologies such as AI-driven monitoring, IoT-enabled wearables, and collaborative robotics intersect with workforce wellbeing. The first approach involves institutionalizing technology-enhanced psychological interventions through standardized mental health protocols. These protocols should utilize digital tools for real-time monitoring of emotional wellbeing while respecting individual dignity. Project managers need to create comprehensive guidelines that include: (1) AI-assisted psychological assessment criteria aligned with safety performance metrics in BIM systems; (2) required regular check-ins using wearable biofeedback devices; and (3) blockchain-secured confidentiality measures for managing emotional data. The second approach focuses on expanding social networks within the connected worksite framework of Construction 5.0. This can be achieved by developing hybrid physical-digital community interfaces, such as: (1) virtual reality programs that help migrant workers connect with local communities through immersive language and cultural simulations; (2) smart project management systems that facilitate skills-sharing initiatives between construction teams and residents using blockchain-verified micro-credentialing; and (3) AR tools that enhance cross-cultural communication during collaborative tasks, such as holographic visualizations of pipe systems with multilingual annotations.

As shown in Tables 2, 3, safety participation is crucial for effective resource allocation at the individual level in Construction 5.0. To improve safety participation, three key strategies are proposed. First, the adaptive safety training system should be redesigned using digital twin technology. Project managers can develop AI-driven training modules that adjust content in real time based on biometric data from IoT-enabled wearables, such as fatigue levels detected by smart helmets. Additionally, integrating knowledge graph technology into BIM platforms can create safety tutorials tailored to specific project risks and current safety standards. Second, a practical training program should be developed that aligns with the new system. This includes creating AI-powered learning profiles to identify each worker's competency gaps and using dynamic content recommendation engines to deliver microlearning modules tailored to individual needs. Third, augmented reality (AR) training interface should be designed to support the safety management system and the training program. The interface will provide personalized safety guidance during equipment use and assess workers' learning performance by comparing real-time actions with safety benchmarks. This approach allows for adaptive feedback and continuous improvement of skills and training effectiveness.

In Construction 5.0, effective leadership safety behavior is essential for meeting workers' safety needs, as detailed in Table 4. To foster a strong safety culture and enhance overall practices, construction managers should implement policy interventions. This includes utilizing AI-driven management, IoT-connected devices, and automated processes within established policy frameworks to efficiently address safety factors. To create a safety-conscious environment in the digital landscape of Construction 5.0, several strategies can be employed. These include establishing a virtual safety hub for sharing safety information and discussing equipment operation, implementing incentive programs that reward individuals for reporting hazards or adhering to safety protocols, and conducting regular safety audits and inspections. Given the high-tech and physically demanding nature of Construction 5.0, safety protocols must be supported by clear project policies and require increased oversight from team leaders. Additionally, to improve the wellbeing of construction workers in this challenging environment, measures such as collaborating with local hospitals, utilizing digital health records, and conducting regular health check-ups can effectively address issues related to fatigue, stress, and mental health. This strategy creates a health monitoring system for both physical and mental well-being, thereby improving overall safety management in Construction 5.0.

## 8 Conclusions and limitations

This study applied the JD-R theory to examine how psychosocial support, safety participation, and leadership safety behavior influence safety practices in the context of Construction 5.0. Our findings revealed that: (1) psychosocial support positively affects both safety participation and safety practices; (2) safety participation contributes positively to safety practices; (3) safety participation fully mediates the relationship between psychosocial support and safety practices; and (4) leadership safety behavior positively moderates the connection between safety participation and safety practices. These findings delineate the mechanisms through which emotional arousal and organizational interventions operate within Construction 5.0, establishing operational principles for strategically allocating emotional resources across dynamic project teams. These results clarify how emotional factors and organizational interventions function within Construction 5.0, providing a framework for effectively allocating emotional resources among dynamic project teams. This research also extends the JD-R theory by demonstrating its relevance in highly connected work environments, where advanced technology-driven analytics require a balance between technological integration and the management of human emotional capital. Practically, the findings offer a guide for developing hybrid management systems that align emotional state monitoring with adaptive leadership strategies. This approach ensures that human-centric safety measures evolve in tandem with the needs of temporary project organizations.

The findings of this study should be viewed in light of its limitations. First, while the research effectively identifies emotional resource depletion among construction workers in Construction 5.0 environments, it does not fully explain the psychophysiological pathways through which regulatory compliance incurs emotional costs. Second, although the focus on China's intelligent construction sector offers valuable insights specific to that context, the findings need to be cross-validated in regions with different regulatory frameworks that are adopting Industry 5.0 principles. Third, although the proposed emotional management framework is conceptually robust, it needs empirical testing with varied samples from different countries or regions to enhance its applicability across cultures and improve result generalizability.

## Data Availability

The raw data supporting the conclusions of this article will be made available by the authors, without undue reservation.
